# Benefits of Regional Integration: Redefining the Concept

**DOI:** 10.1134/S1019331622080020

**Published:** 2022-06-29

**Authors:** O. V. Butorina, Yu. A. Borko

**Affiliations:** Institute of Europe, Russian Academy of Sciences, 125009 Moscow, Russia

**Keywords:** regional integration, regionalism, European Union, customs union, single market, economic and monetary union

## Abstract

For decades, the topic of regional integration has been considered in research through its economic benefits. The classic theories of customs unions and optimum currency area, together with, to a lesser extent, the new regionalism approach are based on analyses of potential costs and benefits of regional associations. Regional integration is traditionally understood as a way to encourage trade flow between member states, to facilitate more efficient allocation of resources by stimulating competition by increasing the capacity of the internal market. That is expected to result in faster economic growth and, consequently, increased per capita income. The 70-year history of the European Union provides sufficient research material to analyze whether economic benefits truly are the main driving force of regional integration. With this goal in mind, the presented article first sums up the key reasons for the post-war unification of Western Europe, then explores the position of the cost-benefit analysis in the theory of regional integration, and finally analyzes the degree of influence of welfare effects on strategic decisions of the European Union. The analysis shows that economic gain is not an immanent property of regional integration: it does not occur at all stages of the process and is neither its primary goal nor its driving force. Instead, regional integration aims to respond to the changing global order, i.e., help member states strengthen their international position and protect themselves from undesirable external influence. While economic benefits are also important, they are not necessarily the decisive factor.

## INTRODUCTION

July 23, 2022 will mark the 70th anniversary of the Treaty establishing the European Coal and Steel Community (ECSC), signed in 1951 in Paris by the following Western European countries: France, Italy, West Germany, and Benelux. This marked the beginning of a new era in the development of Europe: the era of European integration. In 1957, the same six countries established the European Economic Community that was later transformed into today’s European Union of 27 member states and a wide range of integration directions. Having gone through a series of trials over the seven decades of its existence, the European Union remains one of the key centers of power in the modern world. Its experience has become the subject of critical reflection in other regions of the world that are also developing new models of regional integration in response to the current global realities.

The most important tool for evaluating the effectiveness of any integration project is cost-benefit analysis, performed both for the association as a whole and for its individual members (Palankai, Miklos, 2017; Pelkmans, 2013; Burk, Leuffen, 2019; Taghizadeh-Hesary, 2019; Andronova, 2016). The established opinion in political and scientific discourse is that integration should always bring a net gain. This criterion is applied most strictly to associations of developing economies, including post-Soviet countries. The understanding is that countries will not want to unite for the sake of achieving common benefits if the individual gain of each of them does not outweigh the potential losses (Krapohl, Vasileva-Dienes, 2019; Yoo-Duk Kang, 2016; Auriol, Biancini, 2013). To the best of our knowledge, the existing extensive literature on political motives of integration does not consider the question of whether successful associations with a net negative economic effect can exist.

## ORIGINAL MOTIVATIONS

The formation of Western European integration became possible due to a combination of several factors. Firstly, the world was processing the catastrophic outcomes of the two world wars, in which capitalist Europe was both the initiator and the main victim. After World War I, the world’s first socialist state, the Soviet Union, emerged in Eastern Europe, replacing tsarist Russia; after World War II, the global socialist system was established, encompassing eight European states: Albania, Bulgaria, Hungary, East Germany, Poland, Romania, Czechoslovakia, and Yugoslavia. Relations between France and West Germany (FRG) had a previously unimaginable turnaround. The two “eternal enemies” created a dual locomotive to pull forward an increasingly longer train of uniting Western European states.

Secondly, the history of political and social development was reevaluated. Democratic regimes based on the ideas of the rule of law and inalienability of human rights and freedoms had been established in all countries of Western Europe, including monarchies. The state entered into a dialogue with civil society. Spain and Portugal were the last to embark on the path of democracy, having been freed from their authoritarian regimes in the mid-1970s. In the social sphere, Marx’s theory of class struggle between labor and capital to be ended with establishment of the dictatorship of the proletariat gave way to the concepts of social compromise and partnership. Dialogue between trade unions and entrepreneurs became the norm, with the state periodically getting involved to arbitrate. Social policy was becoming the leading item of state budgets (Borko, 2001, pp. 160–165).

Thirdly, at that time Western European countries were headed by an assemblage of outstanding political leaders: Charles de Gaulle, Jean Monnet, and Robert Schuman in France; Winston Churchill, Anthony Eden, and Clement Attlee in the United Kingdom; Konrad Adenauer and Ludwig Erhard in Germany; and Giulio Andreotti and Palmiro Togliatti in Italy. Despite all the differences in their views, they were united by the era and their life experiences. Most of them were witnesses, and many were participants in both world wars and could compare how much bloodier and more destructive World War II was compared to World War I.

Therefore, these political leaders, as well as others, were united by a shared goal: to end the strife between European states by uniting them into a regional organization that promotes cooperation, the peaceful resolution of conflicts, and protection of their interests in the international arena. The ideas of pan-European unity resounded with renewed force: Europe needs to unite to ensure its survival in a world increasingly dominated by the United States and the Soviet Union (Haas, 1997, p. 321).

The first to call for the creation of a united Europe was Winston Churchill, the former Prime Minister of the United Kingdom and a member of the wartime “Big Three” (together with I.V. Stalin and F. Roosevelt). He dedicated his inaugural speech, delivered in September 1946 at the University of Zurich on the occasion of receiving a honorary doctorate, to the “tragedy of Europe”: the consequence of a “series of frightful nationalistic quarrels” between European states. The first step towards reestablishment of the European family was to be a partnership between France and Germany. In the same speech, Churchill proposed to create a federal system in the spirit of the old-established concept of the United States of Europe.^1^

This goal was discussed at the Congress of Europe, held in May 1948 in the Hague. The planned regional organization was to have a charter, the right to create laws, and a system of authorities empowered to implement domestic and foreign policy within their competences. Decisions were made, but almost all of them remained on paper. European states were unwilling to share national sovereignty with the proposed regional association.

Western Europe owes its breakthrough toward integration to three persons: Jean Monnet, Robert Schuman, and Konrad Adenauer.

In the spring of 1949, Monnet proposed a project to create the Coal and Steel Community between France and Germany, open to entry for other democratic states of Western Europe. Monnet is known as a successful entrepreneur and statesman; after the liberation of France, he developed and implemented the Modernization and Re-equipment Plan for the national economy. The first prominent political figure to support him was French Foreign Minister Robert Schuman, the former Prime minister and by 1949 one of the most influential politicians in the country. Like Monnet, he was a proponent of the United States of Europe concept. As for Adenauer, the Chancellor of the newly created Federal Republic of Germany, the motives for his acceptance of France’s offer are obvious. West Germany was offered a favorable option for acceptance into the family of democratic states. In addition, participation in the planned Community promised considerable economic benefits.

The Community was an innovative project: firstly, in terms of its goals, program, and methods. The Treaty established the abolition of all quotas in coal and steel trade among the member states, a gradual reduction of customs duties until their complete abolition, and the creation of a customs union, as well as a common coal and steel market. Two general funds were created: one for financing the modernization of the coal and steel industries, and the other, social, for retraining and reemployment of workers dismissed in the course of modernization. The 10-year program was completed 1.5 years faster, by the end of the 1950s.

Secondly, the Community was a new type of association. Instead of the traditional principle of interstate cooperation of completely independent participants, it was based on an agreement to partially delegate sovereign rights to supranational bodies, making their decisions binding for all member states. The ECSC was first referred to with the term *integration* right after its creation, in 1949. The term was coined by Paul Hoffman, US Administrator of the Economic Cooperation Administration, the body responsible for distributing the funds granted under the Marshall Plan. On October 31, 1949, at the 75th meeting of the Council of the Organisation for European Economic Cooperation (OEEC), he called on the countries of Western Europe “to work towards greater union and to integrate their economies within a large single European market.”^2^ The term *integration* has since then become a regular part of the economic and political lexicon, and the process of the unification of Western Europe has become known as the development of economic integration.

A direct result of the activities of the OEEC and Hoffman’s appeal was the creation of the European Payments Union (EPU) in 1950. It united 17 Western European countries,^3^ also including a significant part of Africa and Asia and some territories of Central and South America, which at the time belonged to Western European currency areas—mainly the pound sterling and the French franc. Over the eight years of its functioning, the EPU fulfilled its objectives: the member states restored convertibility of their currencies, abolished currency restrictions, and eliminated most trade barriers. All of that enabled them to dramatically boost trade, both among partner countries and with third-party countries. There was good reason for Robert Triffin to count the EPU among the forms of economic integration (Triffin, 1956).

Although the European Payment Union included the entire original ECSC six, the experience of its functioning is now being hushed up, and it is resolutely removed from official narratives about the history of European integration. One explanation for that is the fact that the OEEC was built on the principles of interstate cooperation and did not imply the creation of supranational structures (Bulmer, 2007, p. 11). Other reasons can only be a matter of speculation.^4^ However, the EPU had done an impressive job of trade liberalization. By January 1950, quantitative restrictions had been lifted from 90% of imports (Shish-kov, 2001, p. 164). By the end of 1958, all OEEC member states (except Iceland and Turkey) had exempted 80‒98% of transactions in their mutual private (non-public) trade from discriminatory measures (Kaplan, Schleiminger, 1989, p. 344). Therefore, the customs union established by the Treaty of Rome was not created from scratch at all, a fact that is not often brought up today.

Immediately after the ECSC Treaty entered into force in 1952, its members, with the support of NATO, attempted to create two new communities: a defense community and a political community. However, that attempt failed. On August 30, 1954, the National Assembly of France rejected the Treaty establishing the European Defence Community that had already been signed by all ECSC members, including the French Government. This put an end to the plans for the European Political Community, the treaty for which was never even created. In the summer of 1955, at a meeting in Messina, foreign ministers of the “inner six” decided to return to the path of economic integration. Work on two new treaties was launched – the treaty establishing the European Economic Community (EEC) and the treaty establishing the European Atomic Energy Community. Both were signed in March 1957 in Rome, and since then the development of the EEC has become the primary direction of European integration.

## EVOLUTION OF UNDERSTANDING OF THE PHENOMENON

As the phenomenon of regional integration established itself and continued to develop, so did the system of its understanding in the professional sphere. The first research efforts set the objectives of delineating the subject area and developing definitions.

In 1950, Jacob Viner published a book about the consequences of creating customs unions. Its descriptions of the effects of creation and diversion of trade flows (the latter is caused by the removal of customs barriers between the association members while barriers for trade with third-party countries remain in force) retain their scientific value to this day. The book was written in line with the free trade theory, which did not prevent the author from raising the question of the potential role of the customs union in solving a broader problem: improving the welfare of the peoples of the world. According to Viner, the customs union is a very dubious and imperfect means for that goal compared to non-discriminatory reduction of trade barriers worldwide (Viner, 1950, p. 135). The reference to nondiscriminatory reduction of barriers is relevant: trade liberalization within a closed group of countries means the de facto discrimination of outsiders. Customs unions could facilitate liberalization of international trade in some cases and hinder it in others. The overall result, according to Viner, depended on a number of specific factors and could not be calculated in advance (Viner, 1950, p. 51).

James Meade sought to develop Wiener’s economic views, freeing them from the narrow framework of the free trade theory into the vaster field of the welfare theory. While expressing deep appreciation for Wiener’s work, Meade made an important critical observation: within Viner’s analysis, it is not possible to compare the economic benefits from the establishment of a customs union with the losses caused by trade deviation (Meade, 1955, p. 34). To fill that gap, the author constructed a model in which the demand elasticity is zero and the supply elasticity tends to infinity. After applying the model to different situations, Meade came to the conclusion that making a final judgment regarding the impact of customs unions on welfare is impossible. In some cases they contribute to a more rational use of resources, while in others they do not; it depends on a combination of specific circumstances (Meade, 1955, p. 107).

Richard Lipsey of the London School of Economics challenged the assumption that trade creation always has a positive effect on welfare, and trade diversion, a negative one. (Lipsey, 1957, p. 41). The model he developed made it possible to assess the effects of the customs union with greater accuracy and revealed that it results in trade deviation exceeding trade creation; i.e., the total volume of international trade is reduced. It was theoretically substantiated that a situation in which such an alliance raises global welfare is possible. However, naturally, situations with the opposite outcome are also not excluded (Lipsey, 1957. pp. 44, 46). Jagdish Bhagwati later clarified Lipsey’s argument and confirmed his general conclusion (Bhagwati, 1971).

Jan Tinbergen understood integration as the creation of the most desirable structure of the international economy, the removal of artificial obstacles preventing its optimal functioning, and the deliberate introduction of desirable elements of coordination and unification. He paid considerable attention to the problem of centralization and decentralization and determining the adequate degree of regulation of international exchanges. In general, he considered integration as part of the more general problem of the optimum economic policy of international relations between independent states (Tinbergen, 1954, pp. 15‒16, 95).

Tinbergen proposed the now textbook division of integration into negative and positive. Negative integration removes obstacles to interaction, for example, by abolishing import duties. Positive integration creates a new spatial quality, for example, by introducing a common customs tariff (Tinbergen, 1954, pp. 76–79). The author did not study the possible impact of integration on welfare. He mentioned welfare only in the context of the general goals of economic policy, focusing on the issues of the efficient use of resources and ensuring equilibrium production and fair distribution of income (Tinbergen, 1954, p. 104).

Herbert Giersch was the first to theoretically substantiate the existence of spatial effects of creating an economic union between several countries. Using examples, he convincingly showed the presence of a gravitational movement of production factors from the periphery, that is, from the outer borders of the association, towards its central regions. According to Giersch, customs borders restrain the forces that facilitate industry agglomeration throughout the world and in Western Europe in particular. The removal of such barriers within a Western European union would increase the concentration of production and population in its industrial center. In general, the formation of extensive free trade zones could increase discrimination against individual regions. This raised the issue of the need to introduce economic policy measures to neutralize the unjustified privileges of central regions and additional burdens on the periphery (Giersch, 1949, pp. 93‒97).

Maurice Byé researched how the customs union can influence the system of international division of labor. He showed that the removal of trade barriers leads to changes in the specialization profiles of the member states under the pressure of competition. A union between countries with complementary specialization profiles is the most favorable option, while a union between countries with the same type of specialization is associated with difficulties. In some cases, the solution can lie in the partner countries transitioning to more specific specializations within the competing industries. In other cases, such a union may create prerequisites for industry degradation in the countries that are relatively poor in money and resources (Byé, 1950, pp. 135, 148).

Both researchers analyzed shifts in the organization of the economic life of a region caused by the establishment of a customs union. It is noteworthy that even before the creation of the ECSC and the EEC, they managed to arrive at a general idea of costs and benefits of integration that was later confirmed in practice. The EU experience has shown that consolidation of the economic space can exacerbate regional imbalances. To smooth them out, the European Union has been implementing various tools and funds of regional and structural policy since the 1970s. However, even today there is no single, generally accepted methodology for calculating economic costs and benefits of integration (Kondrat’eva, 2020, pp. 23‒28).

A major milestone in regional integration studies was the work of Hungarian-born American economist Béla Balassa (Balassa 1961a, 1961b). According to the author, even in 1961, ten years after the signing of the ECSC Treaty of Paris, the concept of economic integration did not have a clear definition in the literature. Some scientists understood it as the removal of all barriers to economic activity, including barriers within national economies, while others considered it only as a term of international relations. Balassa himself welcomed Tinbergen’s understanding: associating the removal of barriers to the movement of production factors with the need for effective economic policy (Balassa, 1961b, pp. 1–3).

Within the public discussion of the time, Balassa reflected on the interaction of market forces and the state policy. He argued that integration required an active state policy: to maintain full employment, counteract negative spatial effects of integration, regulate cartel activities, and harmonize the national policies of the member states. This position did not prevent him from being critical of integration recipes of French dirigists (Balassa, 1961b, pp. 9, 10).

The author of the classic concept of regional integration stages could not avoid the question of its effect on public welfare. However, Balassa’s scientific contribution lies not in solving the problem but in a comprehensive study of economic effects of integration that may eventually affect welfare (Balassa, 1961a, pp. 10–14). He supplemented Viner’s static integration effects with two more categories: dynamic growth effects (due to the scale effect and the increasing competition) and effects of geographical distribution of production and income within a regional association (Sapir, 2011, pp. 1207, 1208).

Although Balassa’s book marked a turn in the development of the theory of economic integration, it took a while for its ideas to become a part of the political discourse. For example, Jean Monnet in a 1963 article does not mention economic benefits of integration at all. The “father of Europe” recalls that during the war Western European countries suffered enormous damage, at the time of its end they were divided, and within a decade-and-a-half some of them had lost their empires. However, the “loss of their former greatness and prestige” did not make them give up; on the contrary, they were creating the Single Market, which in the future may lead to the creation of a European Federation (Monnet, 1963, pp. 204, 208). The value of the EEC, according to Monnet, lies in the fact that it gives the continent a market comparable in scale to the American market (Monnet, 1963, p. 205). While at the moment no European country can really influence international processes, united they will obtain the political influence necessary to interact with the United States on an equal footing. Monnet concludes that a European union is not a theory but an ongoing process of peoples and countries uniting in order to adapt to the changing global circumstances together (Monnet, 1963, pp. 209–211). Thus, Monnet saw integration as primarily an instrument of collective integration into a new geopolitical reality.

## THE CONCEPT OF BENEFITS IN INTEGRATION PRACTICE

Over its 70-year existence, European integration has passed through several stages. Their goals, content, and results have been thoroughly described in the foreign and Russian scientific literature. The aim of this article is to find out how the theoretically postulated benefits of economic integration have manifested in practice at various stages of the development of the European Union.

The first period (from the creation of the ECSC to the end of the 1960s) was marked by recovery growth, low unemployment, and the gradual expansion of the social functions of the state. The integration project was also developing dynamically. In 1962, the EEC introduced the Common Agricultural Policy with a unified market of agricultural commodities. A customs union that abolished duties and quotas on trade in industrial goods was established in the summer of 1968. Thus, the major advancements of that stage belonged to the sphere of negative integration. The achieved economic benefits resulted from the removal of barriers, while the effects of integration on trade and trade deviation were often accompanied by its general expansion, in line with the global trend.

The second stage (from the early 1970s to the mid-1980s) was characterized by inactive integration dynamics. The union was going through a period of stagnation and “Eurosclerosis.” The governing bodies of the EEC sought to prevent disintegration, spending most of their efforts on maintaining the project in a viable state. Individual positive shifts did not concern welfare effects in any way. During this period, the European Council was created, the European Monetary System emerged, and the implementation of regional and scientific and technical policies began.

The economic benefits direction became central at the third stage, after Jacques Delors became the President of the European Commission in 1985. The Single Market Programme (SMP), implemented by 1992, was based on ideas about the advantages of a large economic space. The EU countries expected to receive long-term benefits from its creation: accelerated economic growth and increased competitive ability through increased competition, economies of scale, and more efficient use of resources. That no longer concerned only static integration effects but also dynamic ones. The potential of the SMP was to be fully realized through the creation of the Economic and Monetary Union. The introduction of a single European currency in 1999 saved market participants from conversion costs, increased price transparency, lowered interest rates, and increased the predictability of economic conditions.

The fourth stage was marked by the expansion of the European Union to the east, when between 2004 and 2007 it accepted 12 new member states,^5^ ten of which were considered part of the Central and Eastern Europe (CEE) region. They were different from the countries that had already been part of the European Union in that their economies were less advanced and their recent experience consisted of almost half-a-century of Soviet-style socialism. The economic consequences of such a large-scale expansion were interpreted in different ways both during the lead-up and now, almost two decades later.

An important note: the extensive scientific literature available on this topic can be divided into two clearly separate parts. The first one examines the economic issues of the new members (Eastern countries); and the second one, the consequences for the old (Western) composition of the European Union. The first group of countries was facing a difficult transition from a centrally planned economy to a market economy, liberalization of external relations, and adaptation to high competition in the EU single market. They expected the reward of an influx of Western investments and technologies, guarantees of economic freedoms, a decrease in inflation, and an increase in welfare. The latter group received an additional 100 million consumers for the EU market and the extension of the regulatory force and values of the European Union to the former buffer zone between the West and Russia. Their costs were associated with the overall manageability of the union and its decision-making mechanisms, financial costs, and the risk of an influx of migrant workers from the new member states. To the best of our knowledge, no studies have examined the economic costs and benefits of the expansion for the entire European Union as a whole, with the exception, at a stretch, of studies on the changing role of the expanded European Union in the global economy and its trade and economic relations with third-party countries, for example, Russia (Ivanov, 2006).

What is the outcome of the new members’ participation in the European Union, for them and for the entire association? What was achieved and which hopes did not materialize?

On the one hand, the CEE countries have clearly benefited in terms of welfare gains. While in the EU nine,^6^ the GDP per capita in 2019 was 103–160% of 2005 values (on average, 126%), in the CEE countries, the rates of its increase were drastically faster. The 2019 per capita GDP of six of them—Bulgaria, Latvia, Lithuania, Romania, Slovakia, and Estonia—were more than double the corresponding 2005 values, ranging from 212% in Slovakia to 280% in Romania. For Slovenia, the Czech Republic, Hungary, and Poland, the per capita income in 2019 was 144–197% of the 2005 level.^7^ The CEE countries were deeply integrated into intraregional trade; for many of them their EU-27 partners now account for 70% or more of their total foreign trade turnover. However, the process of reorientation of their trade flows from partners in the former Comecon to the EU countries had taken place back in the 1990s and was mostly completed by the time they officially joined the union. Moreover, in that process trade deviation was almost always accompanied by trade creation (Feit, 2002, pp. 309, 316; Khesin, 2003, p. 45).

Meanwhile, the economic connectivity of the CEE region itself still remains relatively low. Thus, investment cooperation of the Visegrád Group of countries is hindered by the fact that their national economies are based on small and medium-sized enterprises, which have difficulty competing with Western European TNCs (Gabarta, Drynochkin, 2017, p. 73). Some researchers, including Western ones, point out persistent differences in the market economy models of Western and Eastern Europe. Underpinned by national specifics, these differences may increase in the conditions of globalization, consolidating the subordinate position of the CEE countries in relation to the EU core (Lobanov, Glinkina, 2020; Butorina, 2017, p. 39). In this context, the issue of the cost-benefit ratio of economic integration acquires a new dimension.

The global financial crisis of 2009 and then the European debt crisis revealed many previously unnoticed risks of the single currency. After that, the understanding of costs and benefits of economic integration expanded and shifted towards the former. Around the same time it became obvious that two long-term programs of socio-economic and technological development of the European Union—the Lisbon Strategy and the Europe 2020 strategy—had failed. The resources of integration as an incentive for economic development had apparently been exhausted.

Many researchers note that the biggest challenge the European Union faces today is geopolitical and geo-economic in nature. The European Union’s share in the world’s population and GDP is steadily decreasing, the main center of global development is moving to Asia as the role of the United States weakens. The decentralization of globalization, the political and financial instability, and the fluidity of the ideological framework require the European Union to rethink its integration strategy (Kaveshnikov, 2021, p. 346; Lavery, Schmid, 2021, p. 1322). In recent years, the European Union has taken a decisive step away from the economic agenda. The new key directions are universal: digitalization, the European Green Deal, ensuring external stability of the European Union, and responding to the COVID-19 pandemic. Of course, each of these four vectors has a significant economic component. Digital and green technologies are designed to help society and the economic system transition to a new, more progressive model of production and consumption. The question of the ratio of costs and future benefits of that transition is currently the subject of extensive discussion both in the media and among professionals. Combatting the pandemic also requires major investments.

This part requires an important clarification. Of course, integration projects of the modern European Union can and should be analyzed in terms of their economic cost-benefit balance. However, that analysis should be done with the understanding that achieving an immediate increase in welfare is not the primary goal of these projects the way it was in the case of the customs union; instead, they are aimed at solving a more complex objective that cannot be analyzed in accounting terms. The European Union currently faces the challenge of proving whether it has enough strategic prospects and can survive in the new geopolitical context. Responding to that kind of challenge primarily involves avoiding external threats and minimizing the inevitable damage rather than obtaining future benefits, which is typical for other regional associations elsewhere in the world as well (Butorina, 2021). The situation was similar in the 1950s, when the EEC countries united in order to find their place in the new conditions of bipolar confrontation and the collapse of the colonial system, as explicitly stated by Jean Monnet.

## CONCLUSIONS

The original motives of EEC founders lay mainly in the field of geopolitics and were brought on by the new situation that had formed in the world and in Europe after World War II. Receiving economic benefits from integration was a secondary objective treated as part of the liberal ideology and the global fight against protectionism.

The concept of economic benefits of integration was formed in the 1950s–1960s, mainly as part of the customs union theory. The abolition of customs barriers contributes to the development of intraregional trade with positive consequences for welfare. According to this theory, it is not possible to assess the overall outcome of trade creation and deviation *ex ante*. Nevertheless, the existing consensus is that the creation of the customs union in the European Union in the 1960s lead to an overall positive result from negative integration measures, i.e., from static integration effects.

Dynamic integration effects associated with the increasing capacity of the single market, increasing competition, and better use of space were the base for the strategy of creating the Single Market and the Economic and Monetary Union. Negative integration measures (abolition of barriers to the movement of services, capital, and persons, removal of administrative and technical obstacles) were combined with positive integration measures, including the introduction of a single currency. However, as integration progressed many of its previously unknown costs were revealed, including those related to structural features of national economies. Therefore, the material benefits to costs ratio of integration remains a matter of faith rather than convincing calculation.

Throughout the history of European integration, it has pursued important geopolitical goals that have little to do with the concepts of benefits and costs. The current strategic priorities of the European Union do not include the objectives of maintaining sustainable growth, improving welfare and social cohesion, or economic issues in general. Therefore, using assessments of net benefits of integration as the only and main measure of its effectiveness when analyzing integration processes outside Europe seems unreasonable and not relevant to reality.

This does not contradict the fact that member states of numerous international free trade agreements, the vast majority of which are bilateral, receive economic benefits. However, multilateral integration associations with more complex structures set and solve more complex strategic objectives. The subject of their activity is (to varying degrees) the global position of the region, and that cannot be effectively analyzed by measuring profits and costs.

## Footnotes

1 The United States of Europe. https://churchill.pw/soedinennye-shtaty-evropy.html. (Cited February 2, 2019).

2 Statement by the E.C.A. Administrator at the 75th Council meeting Paris, 31st October, 1949. https://www.cvce.eu/content/publication/2009/4/3/840d9b55-4d17-4c33-8b09-7ea547b85b40/publishable_en.pdf. Cited September 9, 2021.

3 Austria, Belgium, the United Kingdom, Greece, Denmark, Ireland, Iceland, Italy, Luxembourg, the Netherlands, Norway, Portugal, Turkey, France, West Germany, Switzerland, and Sweden.

4 There are several potential reasons why the EPU may be inconvenient for the chroniclers of the European Union. Firstly, to better identify itself with Europe, the European Union desires to “weed out” the history of European integration to exclude unrelated phenomena, such as the European Free Trade Association, the Council for Mutual Economic Assistance, the activities of the Council of Europe, and even integration projects in Northern Europe. Excluding them and the EPU makes the European Union seem like the mainline trend with a clear evolutionary logic: development from simple to complex forms accompanied by spatial expansion. The second and probably main reason is the leading role played by the United States in the formation of the EPU, meaning that it practically forced the OEEC countries to embark on the path of integration. The third reason is the somewhat colonial nature of the EPU that *de facto* included colonies, protectorates, and other dependent territories of the Old World metropoles. In modern political rhetoric, this is particularly unseemly.

5 Bulgaria, Hungary, Cyprus, Malta, Latvia, Lithuania, Poland, Romania, Slovakia, Slovenia, Czech Republic, and Estonia.

6 France, Germany, Italy, Belgium, the Netherlands, Luxembourg, the United Kingdom, Ireland, and Denmark.

7 Data in US dollars at current prices. Source: electronic database UNCTAD-Stat: https://unctadstat.unctad.org/wds/TableViewer/tableView.aspx. Cited September 28, 2021.
